# Innovations in Antibody-Drug Conjugate (ADC) in the Treatment of Lymphoma

**DOI:** 10.3390/cancers16040827

**Published:** 2024-02-18

**Authors:** Ali Al Sbihi, Maryam Alasfour, Georgios Pongas

**Affiliations:** Sylvester Comprehensive Cancer Center, University of Miami, Miami, FL 33136, USA

**Keywords:** Antibody–Drug Conjugate (ADC), linker, payload, Hodgkin Lymphoma, Non-Hodgkin Lymphoma, follicular, DLBCL

## Abstract

**Simple Summary:**

Antibody–Drug Conjugate (ADC) is a novel therapy that has revolutionized the treatment of human cancers. In this review, we focus on the role of ADC in lymphoid malignancies. We summarize the latest advances of ADC development, and we discuss the landmark and most updated clinical trials.

**Abstract:**

Chemoimmunotherapy and cellular therapy are the mainstay of the treatment of relapsed/refractory (R/R) lymphomas. Development of resistance and commonly encountered toxicities of these treatments limit their role in achieving desired response rates and durable remissions. The Antibody–Drug Conjugate (ADC) is a novel class of targeted therapy that has demonstrated significant efficacy in treating various cancers, including lymphomas. To date, three ADC agents have been approved for different lymphomas, marking a significant advancement in the field. In this article, we aim to review the concept of ADCs and their application in lymphoma treatment, provide an analysis of currently approved agents, and discuss the ongoing advancements of ADC development.

## 1. Introduction

Lymphomas are a heterogenous group of malignancies that result from the clonal proliferation of different types of lymphocytes (B cells, T cells, or Natural Killer (NK) cells) at varying stages of their maturation [1]. The incidence of different types of lymphoma has been increasing over the last few decades [2]. Chemoimmunotherapy and cellular therapies remain the commonly used treatment lines for different subtypes of lymphoma [3,4,5]. Most chemotherapy options have a narrow therapeutic index resulting in a wide range of toxicities [6,7]. Additionally, emergence of resistance is among the most encountered reasons precluding its use in the relapsed and refractory (R/R) setting [8,9,10].

Monoclonal antibodies against surface antigens such as Rituximab and Obinutuzumab have revolutionized the treatment of lymphomas [11,12,13,14]. Antibody–Drug Conjugates (ADCs) are highly targeted drugs that link a monoclonal Antibody (mAb) against a specific surface antigen to a cytotoxic molecule. The toxin is delivered only to the cells that express the surface antigen, conferring high tumor specificity with limited systemic exposure [15]. The Food and Drug Administration (FDA) has approved three ADCs for the treatment of lymphomas, which are Brentuximab Vedotin, Polatuzumab Vedotin, and Loncastuximab Tesirine [16].

In this article, we review the concept of ADCs, focusing on their structure, engineering, and role in lymphoma treatment. Additionally, we provide a comprehensive analysis of the currently approved ADCs in this setting, along with an overview of ongoing advancements. This includes highlighting promising results from pre-clinical and clinical trials, as well as discussing the future directions in the development of the next generation of ADCs.

## 2. Basic Structuring of ADC

An ADC is composed of an antibody, drug (payload or cytotoxin), and a linker. The antibody binds a specific antigen that is expressed on the surface of malignant cells. Once attached, the ADC is internalized and the cytotoxic payload is released into the cell, causing cell cycle termination and apoptosis [17]. The cytotoxic effect is also seen in the surrounding target-negative cells, which occurs when the drug diffuses into them and causes “bystander killing”, which is a type of cell death that occurs when the payload is released from the target cell after the ADC is internalized and degraded or when the drug is released into the extracellular space [18]. Notably, for better results and fewer side effects, the binding between antigens and antibodies needs to be as specific as possible [19]. The internalization rate of the payloads, the stability of the linkers, and the choice of corresponding antibodies are some of the variables that influence the use of ADCs in clinical practice [20].

### 2.1. Monoclonal Antibodies

The selection of an appropriate antibody is a crucial step in the engineering of an ADC; it is typically designed to specifically bind the target antigen expressed on cancer cells. Ideal monoclonal antibodies should have low immunogenicity, low cross-reactivity, a long plasma half-life, in addition to high binding affinity to the surface antigen of the tumor cell [21]. The most commonly utilized class is immunoglobulin G (IgG), more specifically the IgG1 subtype, due to its serum stability [22].

In the early stages of ADC development, mice-derived antibodies were predominantly employed. However, these were reported to have high failure rates, likely attributable to their lack of clinical benefit while causing serious immunogenicity-related adverse effects. Those ADCs were also rapidly cleared by human anti-mice antibodies limiting their efficacy [23]. Moreover, the linkers utilized in these early ADCs were unstable in human circulation, leading to the premature release of the payload, which substantially reduced effectiveness and increased toxicity [24]. Since the emergence of humanized mice monoclonal antibodies, which have significantly reduced immunogenicity, chimeric and humanized antibodies have replaced murine antibodies leading to more optimal designs [25], such as Gemtuzumab Ozogamicin, the first ADC approved by the FDA in 2000 for patients with CD33-positive acute myeloid leukemia [26]. To date, fourteen ADCs have received market approval from the FDA, of which three have been approved for the treatment of lymphomas, as previously mentioned. Among them, only Brentuximab Vedotin uses a chimeric antibody [27].

Chimeric Abs are designed by fusing domains from different species (e.g., the entire variable region from mice or rabbits with the constant domain of human origin). In contrast, humanized antibodies contain segments of foreign-derived amino acids grafted into human-derived Fab regions and constant regions. Hence, they are less immunogenic and represent safer choices [28]. In fully human antibodies, no part is mouse-derived, leading to a lower incidence in the immune response when compared to the humanized counterparts; an example is the FDA-approved Enfortumab Vedotin [29].

### 2.2. Payload

**Figure 1:**
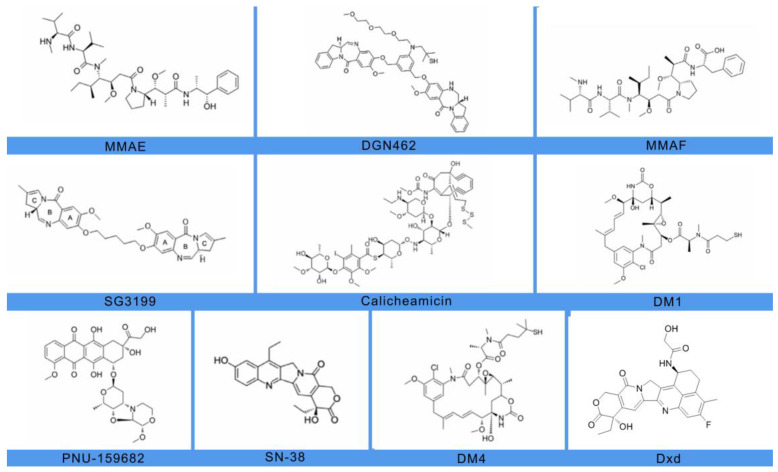
Molecular structures of ten different payloads commonly used in designing ADC.

An ADC’s ideal payload should have distinctive characteristics of low immunogenicity, long half-life, small molecular weight, good solubility in the aqueous environment of antibodies, high stability in the circulation and lysosomes, in vitro sub-nanomolar half maximal inhibitory concentration (IC-50), and functional groups to facilitate the conjugation to the antibody while maintaining the ADC’s internalization property. Each antibody binds to an average of two to four potent cytotoxic molecules [30]. Currently, the two most popular classes of payloads in ADC design are microtubule-disrupting agents and DNA-targeting agents (Figure 1).

Tubulin inhibitors can be classified into two main categories based on their mechanism of action [31]. The first group comprises microtubule-stabilizing agents. These agents enhance filament stability by inhibiting microtubule depolymerization and promoting polymerization, making filaments less functional. The second group consists of substances that destabilize microtubules, preventing tubulin assembly and the development of mature microtubules. The main tubulin inhibitors used and investigated in ADC development are Vinca Alkaloids, Taxanes, Auristatins, Maytansinoids, Cryptophycins, Hemiasterlin, and Discodermolide. Auristatins such as monomethyl auristatin E (MMAE) disrupt microtubules and prevent cell division by preventing α- and β-tubulin monomer polymerization and triggering apoptosis. The binding region is located at the interface between the β1-tubulin and the α2-tubulin subunit of two longitudinally aligned tubulin dimers. According to in vitro findings, auristatin–tubulin bonds form ring- or spiral-shaped aggregates that modify microtubules’ function. Vinca alkaloids, such as Vincristine or Vinblastine, as well as Cryptophycin and Dolastatins, induce a similar curved structural conformation of microtubules [32].

DNA double-strand breakage, alkylation, intercalation, and cross-linking are four main mechanisms through which DNA-damaging agents can kill cancer cells during any stage of the cell cycle. The most commonly used DNA-damaging payloads are the Calicheamicins, Duocarmycins, Pyrrolobenzodiazepine (PBD), Doxorubicin, and Camptothecin analogues [33]. Targeted agents such as BCL-2 inhibitors, spliceosome inhibitors, and transcription inhibitors targeting RNA polymerase II are additional payloads with different mechanisms of action [30,31,32,33,34,35,36,37].

Of notice, PBD payload was noted to have a higher toxicity profile as per an FDA oncology analysis of 15 ADCs containing PBD as a payload, which showed common adverse events included possible vascular leak syndrome, transaminitis, bone marrow suppression, gastrointestinal events, metabolic effects, musculoskeletal events, neuropathy, dyspnea, and kidney injury, all of which result from off-target delivery of PBD; this analysis showed that 47% of investigational new drug applications for PBD-conjugated ADCs between 2013 and 2017 were discontinued [38]. The higher incidence of life-threatening toxicities is a limitation of these highly efficacious ADCs.

Existing mechanisms to mitigate the toxicity of PBD include PEGylation of the drug-linker component, which results in significant improvement tolerability of the ADC [39]. Another mechanism is using the Para-Amino-Benzyloxy-Carbonyl (PABC) self-immolate spacer in the linker-drug; the spacer has a stable disulfide bridge that allows a rapid release of free payload intracellularly, resulting in a better plasma stability and improved toxicity when compared to cleavable linkers [40,41]. The latter study deemed that both the peptide-linked and disulfide-linked PBD ADCs provided similar efficacy in two different lymphoma models with a better safety profile of the disulfide-linked PBD ADC. A third mechanism was the chemical modification of the PBD’s structure by a reduction in one of the imine functional groups, thus changing DNA crosslinking PBD-dimer to a DNA-alkylating metabolite named Indolinobenzodiazepine dimers (IGN) that resulted in a more efficient release of the payload with better efficacy in murine tumor models, where mice tolerated IGN-conjugated ADCs at a higher dose [42,43].

Recent advancements in the payloads have also been under development. The main ones are the immunomodulating ADCs, such as Stimulators of Interferon Genes (STING) agonists, Toll-Like Receptor (TLR) agonists and Targeted Protein Degraders (TPD) using a proteolysis-targeting chimera (PROTAC) strategy [44]. STING agonists activate the production of interferons and other cytokines resulting in augmentation of the immunological and antitumor response [45]. Incoporating STING agonists as payloads in ADC can achieve their delivery to the tumor target with higher efficacy and less toxicity from an excessive inflammatory response due to immune activation [46]. Examples of STING agonists include DMXAA, c-di-AMP, c-di-GMP, cGAMP, ADU-S100, MK-1454, SB11285, BMS-986301, E7766, MSA-1, and MSA-2; they are being mainly explored in breast cancer [47]. TLR agonists improve the innate and adaptive antitumor immunity by enhancing the antigen presentation by antigen-presenting cells in the tumor microenvironment, resulting in more cytotoxic T-cells promotion and activity [48]. They play a role in localized cancers, e.g., BCG (TLR2/4 agonist) for bladder noninvasive carcinoma, AS04 (a TLR4 agonist) for cervical cancer, and imiquimod (a TLR7 agonist) for superficial basal cell carcinoma [49]. Currently, many TLR agonists are being investigated as payloads in ADC in cancer immunotherapy. An example is BDC-1001 (ADC of TLR7/TLR8 agonist conjugated with anti-HER2 antibody), which showed promising results in preclinical models [50,51]. It is currently being investigated in a phase I/II clinical trial that is recruiting patients with advanced HER2 expressing solid tumors (NCT04278144). PROTAC is a catalytic that works on small doses, and it targets a specific protein of interest through ubiquitination-mediated proteolysis [52]. When the PROTAC payload is conjugated to an antibody, it results in a novel combination of PROTAC catalytic behavior and tissue specificity of the antibody, which has a potential for better efficacy and less limitations [53]. Successful examples exist in HER2 expressing cells [54,55]. These delicate improvements in the ADC design have a great potential for better outcomes in oncology through continuous research that can open the door for developments in other cancer types including lymphomas.

### 2.3. Chemical Linker

Linkers are the most important and intricate part in the development of a successful ADC, since they form the link between the therapy and antibody. Their conjugation mechanism, stability, and chemistry are crucial in preventing unintended drug release into the bloodstream and in facilitating easy cleavage upon internalization into the cancer cell, allowing the payload to be released exclusively at the intended site. They are essential in figuring out the ADCs’ pharmacokinetics, pharmacodynamics, and therapeutic window. The currently FDA-approved ADCs use two classes of linkers, which differ in the payload release mechanism: cleavable and non-cleavable linkers [24,56,57].

The four most used cleavable linkers are β-glucuronidase-sensitive linkers, Glutathione (GSH)-sensitive disulfide linkers, cathepsin B-sensitive linkers, and hydrazone linkers. The previously described bystander effect, which impacts normal cells in the tumor microenvironment and tumor cells with no or low target expression, is more likely to occur with ADCs that utilize cleavable linkers [58,59].

**Figure 2:**
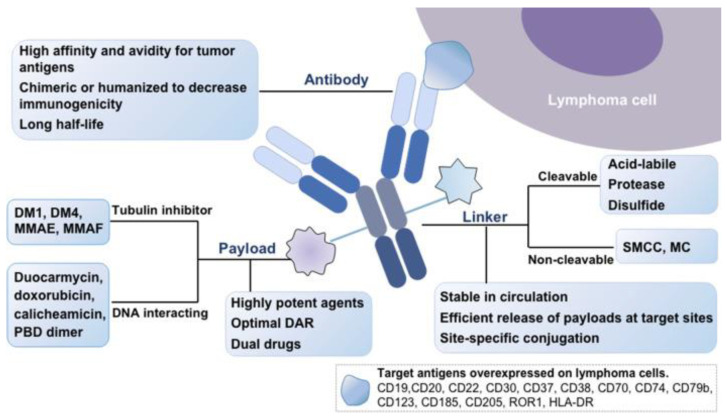
General basic structure of an ADC. An ADC consists of a chimeric or humanized (less immunogenic) antibody that has strong binding activity to an antigen expressed on the surface of a lymphoma cancer cell. Antibodies should have a higher half-life in circulation to be delivered to the target cell surface. Antibodies are linked to payloads of different structures (mainly, tubulin inhibitors or DNA interacting), which are highly effective drugs with ideal drug/antibody ratio. The linkage between the antibody and payload is through cleavable or non-cleavable linkers, which should have precise binding, stability in circulation, and ensure efficient payload distribution at their targets. Reprinted/adapted with permission from reference [62]. Copyright year: 2021, copyright name: Journal of Hematology & Oncology.

Non-cleavable linkers, as opposed to cleavable linkers, have better plasma stability, less off-target toxicity, and resistance to proteolytic degradation, as they have non-reducible bonds with the amino acid residues of the antibody. Generally, non-cleavable linkers are formed by thioether or Maleimidocaproyl groups. Non-cleavable linkers that are attached to an amino acid residue derived from the degraded antibody must have the antibody moiety completely broken down by cytosolic and lysosomal proteases to release the payload following ADC internalization [60,61]. A general basic structure is illustrated in Figure 2 [62].

## 3. FDA-Approved ADCs for Lymphomas

As of 2023, three ADCs have received FDA approval for various types of lymphomas, which are Brentuximab Vedotin (BV), Polatuzumab Vedotin (PV), and Loncastuximab Tesirine (LT) [63], as shown in Table 1.

## 4. Brentuximab Vedotin

**Figure 3:**
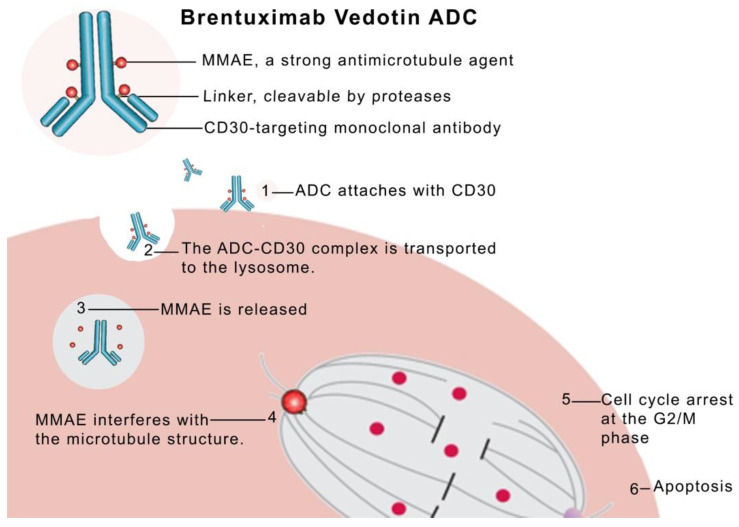
The structure and mechanism of action of BV. It consists of a monoclonal antibody that targets antigen CD30 on a lymphoma cell. After binding, the whole ADC–antigen complex is intracellularly transported to the lysosome where MMAE (payload) is released to interfere with the microtubular structure in the nucleus resulting in cell cycle arrest and apoptosis. Reprinted/adapted with permission from reference [68]. Copyright year: 2012, copyright name: Korean Society of Hematology.

Brentuximab Vedotin (BV) consists of the IgG1 chimeric monoclonal antibody against CD30 “cAC10” and the cytotoxin MMAE, which are bound through a stable linker (Cathepsin-cleavable linkers and Para-aminobenzyl-carbamate spacers) [64]. The chimeric monoclonal antibody cAC10 binds to CD30 followed by endocytosis and subsequent fusion of the vesicle with the lysosomes, where the lysosomal cysteine proteases cleave the linker, releasing MMAE directly into the cancer cell and subsequently resulting in cancer cytotoxicity [65]. The linker system between the Ab and MMAE was designed to be stable in the circulation and is composed of a Maleimidocaproyl spacer, the protease-sensitive dipeptide Val-Cit, the self-immolating PABC moiety, and an additional spacer between the dipeptide and the drug. The latter has the capacity to self-cleave and facilitate cathepsin B access to its cleavage sequence. A thioether bond forms between the maleimide portion and the terminal thiol of the cysteine residues in the heavy and light chains of the antibody, which binds the linker to the antibody [66]. A spontaneous 1,6-elimination of the unstable PABC-substituted MMAE intermediate, produced by the protease cleavage of the Cit-PABC amide bond, yields free molecules of MMAE, CO2, and p-aminobenzyl alcohol [25]. Notably, CD30 is very strongly expressed on malignant lymphoma cells and to a lesser extent on normal tissues [67]. BV can induce growth arrest of CD30^+^ cell lines of up to 340-fold when compared to the antibody alone [65]. The structure and mechanism of action of BV is illustrated in Figure 3 [68].

The malignant Reed–Sternberg cells of cHL are characterized by the expression of CD30, a member of the tumor necrosis factor super-family, and given its restricted expression to a small proportion of activated B cells, T cells, and eosinophils, it represents an ideal target for monoclonal antibody therapy [69]. BV has shown remarkable activity in previously untreated patients with cHL and R/R disease, leading to FDA approvals in these settings.

The ECHELON-1 phase III trial randomized 1334 patients with previously untreated stage III or IV cHL to receive either BV-AVD (Brentuximab Vedotin, Adriamycin, Vinblastine, Dacarbazine) or ABVD (Adriamycin, Bleomycin, Vinblastine, Dacarbazine). The trial showed superiority of the BV-AVD arm with a 2-year modified progression free survival (PFS) of 82% vs. 77% in the ABVD arm, hazard ratio (HR) of 0.77 [70], and a superior 6-year overall survival (OS) of 93.9% vs. 89.4%, with an HR of 0.59 [71]. Based on the results of the ECHELON-1, the FDA approved BV in combination with chemotherapy for the treatment of adult patients with previously untreated stage III or IV cHL. More recently the SWOG S1826 trial was developed to compare BV-AVD vs. N-AVD (Nivolumab, Adriamycin, Vinblastine, Dacarbazine) for pediatric and adult patients with newly diagnosed advanced stage cHL (stage III or IV). The S1826 study demonstrated a superior 1-year PFS of Nivo-AVD (94%) compared to BV-AVD (86%), with an HR of 0.48, and a comparable adverse effect (AE) profile, so it is currently the preferred regimen for front-line therapy of untreated cHL [72].

While younger patients with advanced stage cHL are more likely to tolerate the multi-agent chemotherapy regimens, the elderly population with cHL have suboptimal outcomes due to multiple factors including comorbidities, poor performance status, inability to tolerate full-dose chemotherapy, and increased treatment-related AEs. A multicenter phase II study evaluated the regimen of sequential BV followed by AVD chemotherapy, with subsequent BV maintenance in patients 60 years and older. The study reported a remarkable 2-year PFS rate of 84% with a 2-year OS rate of 93%. This sequential approach increased the cure rates of cHL in this vulnerable population and is the preferred regimen for the elderly with a new diagnosis of cHL [73].

In the R/R cHL setting, BV was initially studied in patients who had failed Autologous Stem-Cell Transplantation (ASCT). BV monotherapy demonstrated an Overall Response Rate (ORR) of 75%, with Complete Remission (CR) in 34%, and a median Progression Free Survival (mPFS) of 5.6 months. Notably, the median Duration of Response (mDOR) for those in CR was 20.5 months [74]. These results led to the first FDA approval of BV for patients with cHL after failure of ASCT or at least two prior multi-agent chemotherapy regimens in those who are ineligible for ASCT [75]. Subsequently, the AETHERA phase III trial included patients with cHL with relapsed refractory disease who had undergone ASCT and had one of the following unfavorable risks, such as primary refractory HL, relapsed HL with initial remission of <12 months, or extra-nodal involvement at the time of relapse after frontline therapy. The AETHERA study demonstrated that early consolidation with BV after ASCT improved the mPFS to 42.9 months vs. 24.1 months with an HR of 0.57 [76,77]. Based on the AETHERA trial, the FDA subsequently extended the approval of BV for the treatment of patients with cHL at high risk of relapse or progression post ASCT consolidation.

Besides the aforementioned FDA approvals, BV is also recommended by the National Comprehensive Cancer Network (NCCN) guidelines in combination with nivolumab for patients with relapsed or refractory cHL in the second line or subsequent therapy. This recommendation is based on findings from the CheckMate 744 phase II study, which evaluated a risk-stratified response-adapted approach involving Nivolumab plus BV followed by BV with Bendamustine for patients with suboptimal responses. The CheckMate 744 study demonstrated that 59% of patients receiving BV and Nivolumab, and 94% of those subsequently treated with BV and Bendamustine, achieved a Complete Molecular Response (CMR) [78]. These results were remarkably superior to the historically suboptimal CMR rates of 60 to 70% from salvage chemotherapy regimens, setting the stage for higher cure rates in the R/R setting. Ongoing trials of BV in cHL are illustrated in Table 2.

### BV in CD30-Positive T Cell Lymphomas

The universal expression of CD30 in Systemic Anaplastic Large Cell Lymphoma (S-ALCL) and the high expression in other subtypes such as Peripheral T-cell Lymphoma-Not Otherwise Specified (PTCL-NOS), angioimmunoblastic T-cell lymphoma (AITL), and Adult T-cell Leukemia or Lymphoma (ATLL) provides a strong rationale for targeting CD30 in T-cell lymphomas [79,80].

A phase II study in R/R ALCL with single-agent BV after failure of at least one combination chemotherapy regimen showed that 86% of patients achieved an objective response, with 57% CR and an mDOR of 12.6 months [81]. The results of this study led to the FDA approval of BV for R/R PTCL. The ECHELON-2 trial was designed to compare the efficacy and safety of BV in combination with Cyclophosphamide, Doxorubicin, and Prednisone (A+CHP) to standard CHOP (Cyclophosphamide, Adriamycin, Vincristine, and Prednisone) for the treatment of previously untreated patients with CD30-positive PTCL. The A+CHP demonstrated superior mPFS of 48.2 months compared to 20.8 months of CHOP, with a comparable safety profile [82]. The 5-year PFS was 51.4% with A+CHP vs. 43% with CHOP (HR 0.7), and the 5-year OS was 70.1% with A+CHP vs. 61% with CHOP (HR 0.72), with a comparable safety profile between the two arms. Subsequent subgroup analyses showed that S-ALCL mainly benefited from A+CHP, whereas the benefit was unclear for the AITL and PTCL-NOS subgroups [83].

Besides PTCL, the role of BV has been explored in Cutaneous T-Cell Lymphomas (CTCL). The ALCANZA phase III trial included patients with primary cutaneous Anaplastic Large Cell Lymphoma (pcALCL) or CD30-expressing Mycosis Fungoides (MF) who had received one prior line of systemic therapy. At a median follow-up of 45.9 months, the trial favored the BV arm with an ORR of 54.7% vs. 12.5% and an mPFS of 16.7 vs. 3.5 months. Similarly, the median time to the next treatment was significantly longer in the BV arm compared to physician’s choice (14.2 vs. 5.6 months; HR 0.27) [84]. Based on the results of the ALCANZA trial, the FDA approved BV for the treatment of patients with pcALCL or CD30-expressing MF who have received prior systemic therapy. Currently, BV is under evaluation in many early-phase trials, which are included in Table 2.

## 5. Polatuzumab Vedotin

**Figure 4:**
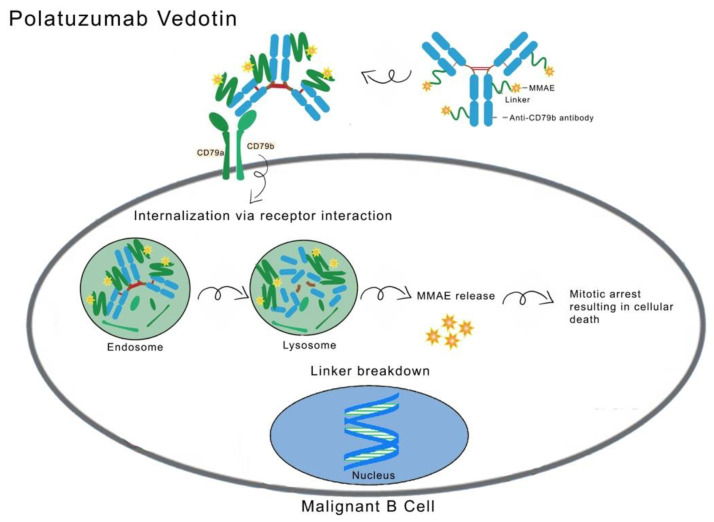
The structure and mechanism of action of PV. PV consists of a monoclonal antibody that targets the antigen CD79b on a lymphoma cell. After binding, the whole ADC–antigen complex is internalized to an endosome and merges with a lysosome. The linker is then cleaved to release MMAE (payload), which in turn results in mitosis arrest in the nucleus and cellular death. Reprinted/adapted with permission from reference [88]. Copyright year: 2020, copyright name: American Society of Clinical Oncology.

The B-cell receptor (BCR) complex is composed of the proteins CD79B, CD79A, and surface immunoglobulin. Notably, CD79B is expressed in more than 90% of B-cell lymphomas, making it a promising target for investigation [85,86]. Polatuzumab Vedotin (PV) consists of an anti-CD79B humanized IgG1 monoclonal antibody bound to MMAE through a Maleimidocaproyl-Val-Cit-PABC protease-cleavable peptide linker. Once Polatuzumab binds to CD79b, it is internalized by endocytosis and directed to the lysosomes, where the linker cleavage occurs, resulting in the payload’s release into the cells to induce apoptosis [87]. The structure and mechanism of action of PV is illustrated in Figure 4 [88].

### 5.1. PV in Diffuse Large B-Cell Lymphoma

Diffuse Large B-Cell Lymphoma (DLBCL) is the most common form of Non-Hodgkin Lymphoma (NHL). While the addition of rituximab to CHOP chemotherapy improved the cure rates substantially, approximately 40–50% of those patients will either have refractory disease or relapse after an initial response [89].

POLARIX was a landmark phase III study that assessed the addition of PV to R-CHP (Rituximab, Cyclophosphamide, Doxorubicin, Prednisone) in the front line setting for DLBCL and compared it to R-CHOP [90]. POLARIX demonstrated a higher 2-year-PFS of 76.7% vs. 70.2%, (stratified HR 0.73) for the pola-R-CHP arm. The overall safety profile was similar in both arms. Notably, the benefit of pola-R-CHP was observed in patients older than 60 years, those with an International Prognostic Index (IPI) between 3 to 5 and in patients with an activated-B-cell like subtype of DLBCL. On the contrary, there was no clear benefit for patients 60 years or younger, those with bulky disease, a lower IPI score or germinal-center B-cell like subtype of DLBCL. Based on the POLARIX trial, pola-R-CHP was the first regimen to demonstrate a superior PFS over R-CHOP in the first line setting for DLBCL and led to the FDA approval for previously untreated DLBCL, Not Otherwise Specified (NOS), High-Grade B-cell Lymphoma (HGBL), or having an IPI score of 2 or greater [91].

The POLAR BEAR phase III trial compared mini-R-CHOP to pola-mini-R-CHP as first line therapy in elderly patients with DLBCL. Initial safety data showed both regimens were tolerable in the elderly (69% were 80 to 90 years) and frail population (16% were frail and 12% had an ECOG performance status of 3), and that the replacement of vincristine with Polatuzumab did not lead to higher incidence of grade 3 to 4 hematologic toxicity or neuropathy. Notably though, there was a higher frequency of gastrointestinal adverse effects (31% vs. 16%) in the pola-mini-R-CHP arm [92]. Based on the results of the POLARIX trial, there is an inclination to hypothesize that pola-mini-R-CHP may demonstrate superior PFS over mini-R-CHOP. Consequently, the outcome of this study is highly anticipated.

In the R/R DLBCL setting, the first trial that evaluated the safety and efficacy of PV was the phase Ib/II trial that compared PV in combination with Rituximab (R) and Bendamustine (B) vs. BR alone for transplant-ineligible R/R DLBCL. The pola-BR arm demonstrated a higher CR (40% vs. 17.5%), a longer mPFS (9.5 vs. 3.7 months, HR 0.36), and a higher mOS (12.4 vs. 4.7 months, HR 0.42) [93]. However, it resulted in higher rates of grade 3 to 4 neutropenia (46.2% vs. 33.3%), anemia (28.2% vs. 17.9%), and thrombocytopenia (41% vs. 23.1%), albeit with similar rates of grade 3 to 4 infections (23.1% vs. 20.5%). Peripheral neuropathy associated with PV was observed in 43.6% of patients, but was grade 1 to 2 and resolved in most patients. This trial led to the initial approval of pola-BR in June 2019 and its broad use in this setting.

Another promising combination of Polatuzumab was demonstrated in a phase Ib/II trial in combination with Mosunetuzumab, a humanized IgG1 bispecific antibody targeting CD20 and CD3 in R/R DLBCL, HGBCL, transformed FL, or grade 3b FL. At a median follow up of 23.9 months, 59.2% of patients responded to Polatuzumab plus Mosunetuzumab with 45.9% achieving a CR. The mPFS was 11.4 months and the mOS was 23.3 months. This regimen was well tolerated, and the most common AE was neutropenia in 25% of patients. Overall, these results showed that the combination of Polatuzumab and Mosunetuzumab can lead to clinically meaningful responses with a favorable safety profile in transplant-ineligible R/R LBCL [94].

### 5.2. PV in Other B-Cell Non-Hodgkin Lymphomas (B-NHL)

Besides DLBCL, combinational therapies that include PV have been explored in indolent B-NHL. In a phase Ib/II trial, the safety and efficacy of the combination Polatuzumab, Obinutuzumab (G), and Lenalidomide (Len) was evaluated in patients with R/R FL. After a median follow up of 27 months, results demonstrated an ORR of 76% with a high CR rate of 63%, although it did not reach the prespecified threshold for activity [95]. After a median follow up of 43.3 months, the mPFS and mOS were not reached, whereas the landmark PFS was 53% at 4 years [96]. These results compare favorably with the available standard of care chemotherapy options in R/R FL and support further investigation of the Pola-G-Len regimen in FL.

Efficacy of PV has also been studied in mantle cell lymphoma (MCL). In a phase II study of R/R MCL, PV was tested in combination with Mosunetuzumab in patients previously treated with two or more prior lines of therapy, including Bruton Tyrosine Kinase inhibitors (BTKi), and demonstrated a high ORR of 75% with a CR of 70% [97]. Nevertheless, more outcome data are needed to assess its role in MCL, where novel regimens are urgently needed for patients refractory to chemoimmunotherapy and BTKi.

## 6. Loncastuximab Tesirine

**Figure 5:**
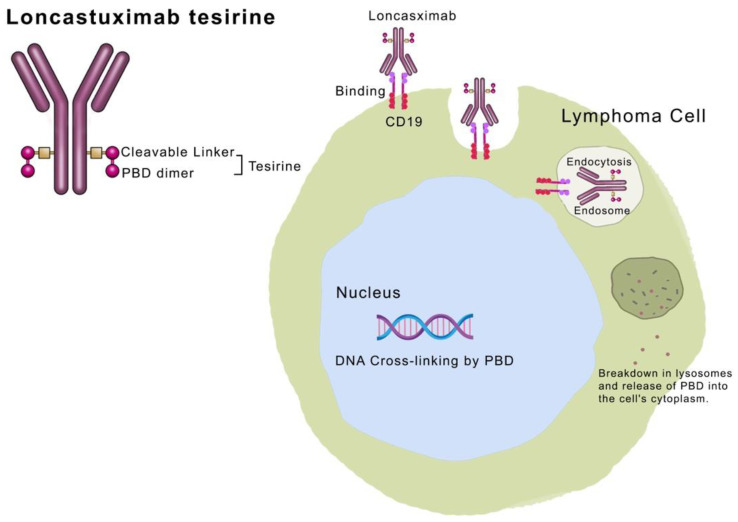
The structure and mechanism of action of LT. LT consists of the monoclonal antibody Loncastuximab linked to PBD dimer (payload) through a cleavable linker (collectively named Tesirine). The antibody binds to the CD19 antigen on a lymphoma cell, after which the whole complex is endocytosed and merged to lysosomes. This is followed by the release of the PBD into the cytoplasm, which travels to the nucleus where DNA cross linking occurs, leading to cellular death. Reprinted/adapted with permission from reference [102]. Copyright year: 2024, copyright name: Touch Medical Media.

CD19 is a surface glycoprotein expressed at the earliest stages of B cell differentiation up until plasma cell development. Besides normal B-cells, CD19 is also expressed in the majority of B-cell lymphomas [98,99]. In contrast to CD20, CD19 exhibits more uniform expression and is retained in small subsets of CD20-negative tumors following anti-CD20 targeted therapy, making it a selective target for immunotherapy and combination therapies [100]. Loncastuximab Tesirine (LT, ADCT-402) is an ADC comprising a humanized anti-CD19 monoclonal antibody conjugated through a cathepsin-cleavable linker to the highly cytotoxic DNA minor groove crosslinking Pyrrolobenzodiazepine dimer toxin (PBD dimer) [101]. Due to its rapid internalization kinetics, trafficking into lysosomes, and stability in the circulation, LT is the most effective ADC targeting CD19. The structure and mechanism of action of LT is illustrated in Figure 5 [102].

### 6.1. LT in Diffuse Large B-Cell Lymphoma

The initial encouraging results of the phase I in-human study, where LT demonstrated clinically meaningful activity in R/R NHL, led to the LOTIS-2 study, which assessed its efficacy in R/R DLBCL [103]. LT demonstrated an ORR of 48.3%, with 50% of responders achieving a CR. While the mPFS was 4.9 months, the mDOR was 13.4 months for those who achieved a CR. It demonstrated a safety profile that was generally well tolerated, with neutropenia being the most common grade 3 AE (26%), followed by thrombocytopenia (18%) and elevated gamma-glutamyl transferase (17%) [104]. The substantial antitumor activity, durable responses, and acceptable safety profile led to the FDA approval in 2021 for management of R/R large B cell lymphoma after two or more lines of systemic therapy. The approval encompassed those with DLBCL NOS, DLBCL arising from low-grade lymphoma and HGBL [105].

Following the landmark study, LOTIS-2, LT was evaluated in combination with other targeted therapies, such as ibrutinib in LOTIS-3 and the monoclonal anti-CD20 antibody, rituximab, against R-GemOX (Rituximab, Gemcitabine, and Oxaliplatin) in LOTIS-5, but it remains unclear if these combinations will move to clinical application (Table 2).

### 6.2. LT in Follicular Lymphomas

LT was evaluated in a phase I study in R/R NHL, where it showed a 78.6% ORR in FL [106]. Very encouraging results were recently presented at the 2023 American Society of Hematology (ASH) meeting from a phase II trial combining LT with rituximab in R/R FL. The trial demonstrated an ORR of 95.2%, which included a CR of 66.7% and a partial response (PR) rate of 28.6% [107].

**Table 2:** Shows ongoing trials in BV, PV, LT.

Regimen	Phase	Disease	N.	ORR	CR	PFS	OS	Grade 3–4 AEs
BV-R-mini-CHP [108]	I	DLBCL ≥ 75 years old Frontline	22	86%	67%	2-year PFS:60.6%	2-year OS: 73.9%	Neutropenia (23%), fatigue (18%), pneumonia (18%),
BV+CHP [109]	II	PTCL with <10% CD30 expression Frontline	55	CD30- 83% CD30 low 74%	CD30- 56% CD30 low 59%	-	-	Total: 53% Febrile Neutropenia (18%)
BV+ICE (Ifosfamide, Carboplatin, Etoposide) [110]	I/II	R/R cHL	45	91%	74%	2-year PFS: 80.4%	2-year OS: 98%	Thrombocytopenia (80%), Neutropenia (73%)
BV+ Romidepsin [111]	I	CTCL regardless of CD30	7	80%	0%	-	-	Fever (14%), Transaminitis (14%)
PV+Obintuzumab+Venetoclax [112]	Ib/II	R/R FL	74	-	57%	22.8 months, 1-year PFS: 73%	-	Neutropenia (39%), Thrombocytopenia (19%)
PV+Rituximab+Venetoclax [113]	Ib/II	R/R DLBCL	48	65%	IRC- assessed: 29% INV-assessed: 31%	4.4 months	11 months	Neutropenia (53%), infections (16%)
PV+R-GemOx vs. R-GemoOx [114]	III	R/R DLBCL	15	40%	27%	-	-	Thrombocytopenia (20%), Neutropenia (13%)
PV+R-ICE [115]	II	R/R DLBCL	38	92% (after 2 cycles) 89% (after 2–3 cycles)	55% (after 2 cycles) 61% (after 2–3 cycles)			Anemia (43%), Thrombocytopenia (43%), Neutropenia (43%)
PV+Rituximab after CART [116]	II	R/R DLBCL	8	50% (All PR)	-	5 weeks	15 weeks	62.5% died
PV+Mosunetuzumab (Bispecific Ab targeting CD20 and CD3) [94]	Ib/II	R/R DLBCL	120	59.2%	46%	11.4 months	23.3 months	Neutropenia (25%), fatigue (6.7%)
LT+Ibrutinib [117]	II	R/R DLBCL	35 GCB: 13 Non-GCB: 22	All: 57.1% GCB: 77% Non-GCB: 45.5%	All: 34.3% GCB: 46.2% Non-GCB: 27.3%	-	-	All: Neutropenia (23%) Thrombocytopenia (17%)
LT+Ibrutinib [118]	I	MCL	7	85.7%	57%	Not stratified according to disease subtype		
LT+Rituximab vs. R-GemoOx [119]	III	R/R DLBCL	20	80%	50%	8.3 months	-	Elevated GGT (25%), Neutropenia (15%)
LT vs. Idelalisib [120]	II	R/R FL	60 Target: 150	-	55% for LT 15% for Idelalisib	-	-	-
LT+Rituximab [121]	II	R/R FL	Estimated: 39	Trial is still accruing				
LT+Venetoclax [122]	I	R/R NHL	Estimated: 36	Trial is still accruing				
LT+R-CHOP [123]	I	First line frail DLBCL	Trial is withdrawn due to sponsor’s decision					
LT+Rituximab [124]	II	First line frail DLBCL	41	Trial is active				
LT after salvage immunotherapy in BTKi-treated/intolerant at R-BAC [125]	II	R/R MCL	Estimated: 56	Trial is still accruing				
LT+PV, LT+Glofitamab, LT+Mosunetuzumab [126,127]	Ib	R/R NHL	Estimated: 200	Trial is still accruing				
LT+Durvalumab [128]	I	R/R NHL	13	Trial is terminated due to limited number and no additional activity for the combination vs. LT monotherapy.				
LT [129]	II	WM after at least 2 prior lines, including an anti-CD20 antibody and BTKi	Estimated: 36	Trial is still accruing				

Abbreviations: GCB: Germinal Center B-cell, BTKI: Bruton Tyrosine Kinase Inhibitors, R-BAC: Rituximab-Bendamustine, Ara-C), WM: Waldenstrom Macroglobulinemia.

## 7. Other ADCs under Study, Development, and Evaluation for the Treatment of Lymphoma

### 7.1. CD19-Targeting Antibody Drug Conjugate

#### 7.1.1. Coltuximab Ravtansine

Coltuximab Ravtansine (SAR3419) is an IgG1 antibody (huB4) linked to the cytotoxic drug DM4 by a disulfide cross-linking agent SPDB that targets CD19 [130]. CD19 is a protein highly expressed in the B lymphocyte lineage at the point of hematopoietic differentiation through pre-B and mature B cells and is maintained in most B cell malignancies [131]. Two phase II trials have investigated the use of Coltuximab Ravtansine as single agent or in combination. An ongoing trial is examining its use in patients with R/R DLBCL as monotherapy, with preliminary data showing an ORR of 44%; however, mPFS, mOS, and DOR have not resulted yet. It has an acceptable safety profile, and the drug was discontinued in 4 of the 41 patients due to adverse events [132]. The other trial examined Coltuximab Ravtansine in combination with Rituximab in patients with DLBCL and showed an ORR of 31.1%, with highest efficacy in patients with relapsed disease (ORR 58.3%), in contrast to refractory disease (ORR 43%) or in the first line setting (ORR 15.4%). Overall, it showed an mPFS of 3.9 months, mOS of 9 months and a DOR of 8.6 months [133].

#### 7.1.2. Denintuzumab Mafodotin

Denintuzumab Mafodotin (SGN-19A or SGN-CD19A) is another CD19 targeting ADC. It is an antibody (hBU12-491) conjugated to MMAF by a non-cleavable maleimidocaproyl linker [134]. A phase I trial that mostly included patients with B-ALL (n = 59), along with aggressive B-Cell Lymphomas (n = 6) and Burkitt Leukemia/Lymphoma (n = 6), showed a DOR of 27 months. It was given every 3 weeks and showed a CRc (CR+CRi+CRp) of 35% in B-ALL. Of the six patients with Burkitt leukemia/lymphoma and the six with B-LBL, only one achieved a CR in each group. The most common AEs were pyrexia (54%) and nausea (52%) [135]. Another phase I trial examining Denintuzumab Mafodotin mainly included R/R DLBCL (n = 53), along with MCL (n = 5) and FL (n = 3) and showed slightly superior activity in DLBCL, compared to the former trial, with an ORR of 33%, CR of 22%, and DOR was 39 weeks for relapsed cases and 41 weeks for refractory cases. The most common AEs were ocular-related: blurry vision (65%) and dry eyes (52%) [136]. Denintuzumab Mafodotin was further studied in combination with chemotherapy in two phase II trials, one combining it with RICE chemotherapy in R/R DLBCL or grade 3B FL, comparing it to RICE alone [137], while the other studied its combination with R-CHOP or R-CHP vs. R-CHOP alone as frontline therapy in DLBCL or grade 3B FL [138]; however, both trials were terminated by the sponsor.

### 7.2. CD22-Targeting Antibody Drug Conjugate

#### 7.2.1. Inotuzumab Ozogamicin

CD22, a 135 kDa type I transmembrane glycoprotein, is a B cell lineage-specific protein expressed on immature and mature B cells. It is upregulated in B cell malignancies including most lymphomas and leukemias [139]. There are two ADCs targeting CD22 currently under investigation in lymphomas. The first is Inotuzumab Ozogamicin (CMC-544), an IgG4 kappa monoclonal Ab with *N*-acetyl-γ-calicheamicin dimethyl hydrazide, a semi-synthetic derivative of calicheamicin linked by 4′-acetylphenoxy-butanoic acid [140], which was FDA-approved in August 2017 for R/R B cell ALL. A phase I trial studied its use in patients with R/R B-cell NHL and showed an ORR of 39% for the 79 enrolled patients, with an ORR of 68% in FL and 15% in DLBCL. However, it had a short DOR with an mPFS of 10.4 months for DLBCL and 49 days for FL. The most common AE was thrombocytopenia in 90% of patients [141]. A Phase II trial for indolent refractory B-cell NHL (n = 81) showed similar results with an ORR of 67%, CR of 31%, and mPFS of 12.7 months [142].

#### 7.2.2. Pinatuzumab Vedotin

Pinatuzumab Vedotin (Pina, DCDT2980S, RG-7593) is an anti-CD22 monoclonal IgG1 antibody conjugated to MMAF by a cleavable maleimidocaproyl-Val-Cit-PABC linker [143]. A phase I trial showed higher efficacy when used as monotherapy compared to its use in combination with rituximab, where the DLBCL group had an ORR of 25% versus 17%, respectively. No objective response was seen in Chronic Lymphocytic Leukemia (CLL) [144]. Its combination with rituximab was further examined in a phase II trial, ROMULUS, showing superior responses compared to R-pola for patients with DLBCL but not with FL; the DLBCL cohort demonstrated an ORR of 60% and CR of 26% on R-Pina (n = 42) compared to an ORR of 54% and CR of 21% on R-Pola (n = 39). The AEs of both were comparable in this trial, with 79% grade 3–5 AEs in the R-pina arm vs. 77% with R-Pola for the DLBCL cohort. The AEs were slightly lower in the FL cohort with 62% and 50% grade 3–4 AEs in R-Pina and R-Pola, respectively [145].

### 7.3. CD25-Targeting Antibody Drug Conjugate

#### Camidanlumab Tesirine

CD25, a type I transmembrane protein and α-chain of the interleukin-2 receptor, is a protein expressed on activated B cells, activated and regulatory T cells, and myeloid precursors and is over expressed in various tumors including lymphomas and leukemias [146]. Camidanlumab Tesirine (ADCT-301, HuMax-TAC) is an IgG1 monoclonal Ab conjugated to PBD dimer warhead (SG3199) by a cathepsin-cleavable Val-Ala dipeptide linker [147]. A phase I trial examined its efficacy in R/R cHL (n = 133) and R/R NHL (n = 56), where it demonstrated a superior ORR of 71% and CR of 42% in those with cHL, compared to R/R NHL (ORR 38% and CR 9%). Grade ≥ 3 AEs included elevated GGT levels (15%), rash (12%), and anemia (11%) [148]. More importantly, 3.8% of patients developed serious neurologic events, including Guillain–Barre Syndrome (GBS)/polyradiculopathy and 27.4% discontinued treatment due to adverse effects. While the CR reported on the study is encouraging, the FDA advised against an approval filing to treat R/R HL, and therefore its future remains unclear.

### 7.4. CD37-Targeting Antibody Drug Conjugate

#### Naratuximab Emtansine

CD37 is a transmembrane protein involved in cell membrane organization and co-signaling that is expressed mainly on mature B cells and to a lesser extent on T cells, macrophages/monocytes, and granulocytes. It is highly expressed in mature B cell malignancies, such as CLL, variably expressed in DLBCL with CD37 positivity ranging from 40% to 90% of cases, and is low or absent in Acute Lymphoblastic Leukemia (ALL) and HL [149]. Naratuximab Emtansine (IMGN529) is an IgG1 monoclonal antibody conjugated to the maytansinoid DM1 by the thioether linker, *N*-succinimidyl-4-(*N*-maleimidomethyl) cyclohexane-1-carboxylate (SMCC) targeting CD37 [150]. Naratuximab emtansine was initially studied in a phase I trial with R/R B-cell lymphomas and resulted in a minimal effect with an ORR of 13% [151]. However, a phase II trial of Naratuximab Emtansine in combination with rituximab, resulted in a higher ORR of 44.7% with a CR of 31.6% in DLBCL and slightly higher response rates in FL with ORR 57% and CR 36%. The mDOR was not reached for the DLBCL and FL subgroup during a median follow up of 15 and 21.8 months, respectively. The treatment was well tolerated overall as demonstrated by improvement in Quality of Life (QoL) measures by three points on average on the Lymphoma Subscale of the FACT-Lym QoL [152]. Overall, it remains unclear whether it will progress to a phase III study.

### 7.5. CD70-Targeting Antibody Drug Conjugate

#### Vorsetuzumab Mafodotin

CD70 is a co-stimulatory molecule and a member of the tumor necrosis factor superfamily that is transiently expressed on antigen-activated B cells, T cells, NK cells, and mature dendritic cells. It is aberrantly expressed in solid tumors and hematological malignancies [153]. Vorsetuzumab Mafodotin (SGN-75) is an ADC that targets the CD70 molecule and microtubule toxin molecule, to Monomethyl Auristatin F (MMAF) [154]. A phase I trial in R/R CD70+ NHL demonstrated unacceptable toxicity, which was mainly thrombocytopenia [155]. Additionally, SGN-CD70A, is an engineered cysteine monoclonal antibody (EC-mAb) conjugated to PBD dimer by a stable, protease-cleavable, peptide-based linker [156], targeting CD70. Similarly, it is also associated with unacceptable toxicity and only modest activity [157].

### 7.6. ROR1-Targeting Antibody Drug Conjugates

#### 7.6.1. Zilovertamab Vedotin

ROR1 is a tyrosine kinase transmembrane receptor expressed in immature B lymphocytes, endocrine glands and the gastrointestinal tract. It is highly expressed in hematological malignancies, especially B-cell lymphomas. Zilovertamab Vedotin (MK 2140 or VLS-101) is a humanized IgG1 monoclonal Ab against ROR1, conjugated to MMAE by a proteolytically cleavable linker [158]. In a landmark phase I trial, Zilovertamab Vedotin demonstrated an ORR of 47% with CR 20% in patients with R/R MCL and ORR 60% in those with R/R DLBCL [159]. The phase II waveLINE-004 study, which utilized Zilovertamab Vedotin as monotherapy for R/R DLBCL, showed slightly lower efficacy with an ORR of 30% with 10% of patients achieving a CR. The most common grade 3-4 AEs were neutropenia (18%), followed by anemia (15%) [160]. Additional studies are currently recruiting; one is a phase II trial combining Zilovertamab Vedotin with R-CHP in the upfront setting for DLBCL and the other is a phase II/III assessing its combination with R-GemOX vs. R-GemOX alone in R/R DLBCL [161,162].

#### 7.6.2. Cirmtuzumab-ADC-7

Another ADC targeting ROR1 is Cirmtuzumab, a monoclonal antibody against ROR1 conjugated to MMAE by a UC-961 linker [163]. In phase I/II, Cirmtuzumab was studied in combination with ibrutinib for the management of R/R MCL or Treatment Naïve (TN) or R/R CLL [164]. In the MCL cohort, Cirmtuzumab demonstrated an 80% ORR with 35% of patients achieving a CR. Notably the mPFS was not reached at a median follow up of 14.9 months. In the CLL cohort, the results continue to be encouraging as they mature and the mPFS has not been reached either at a median follow up of 14 and 7 months for the TN and R/R CLL patients, respectively. Overall, the study is ongoing, and the results appear promising.

### 7.7. ADCs in Pre-Clinical Development

#### 7.7.1. Novel CD30-Targeting ADC: SGN-35C

SGN-35C, while similar to BV in its composition of an anti-CD30 antibody, is distinct in that it is linked to the novel Camptothecin-derived topoisomerase 1 inhibitor payload. Studies conducted in vitro and in vivo on CD30-positive ALCL and HL cell lines, including those resistant to BV, demonstrated treatment induced cytotoxicity. Notably, this effect extends to the CD30-negative cells, indicating a bystander effect. Non-human primate models have also confirmed its safe tolerability. The next phase is human clinical trials, which are currently being planned [165].

#### 7.7.2. CD19 Targeting ADC: IKS03

IKS03 consists of a human-derived antibody targeting CD19, designed for site-specific conjugation of PBD prodrug payloads. It is activated via bioconjugation yielding a conjugate with a drug to antibody ratio of 2. In vitro and in vivo studies have demonstrated effective cytotoxicity in various CD19 positive lymphoma cell lines, most notable in MCL and DLBCL xenograft models that contain genetic aberrations frequently observed in R/R NHL, such as the CCND1 t(11;14) translocation, and triple hit lymphomas, which involve gene rearrangements in BCL-2, BCL-6, and c-MYC. Additionally, it exhibited a lower rate of non-specific payload release from the conjugate compared to other ADCs that employ the PBD pro-drug, including LT [166,167]. A phase I clinical trial, NCT05365659, is currently underway to evaluate the safety profile in patients with advanced B-cell NHLs.

#### 7.7.3. CXCR5 Targeting ADC: VIP924

VIP924 is a first-in-class ADC that targets CXCR5, consisting of a legumain-cleavable linker and a kinesin spindle protein inhibitor (KSPi) payload. CXCR5 is a chemokine receptor that is highly expressed on B- and T-cell-derived lymphomas and is involved in pathways that modulate tumor cell invasion, growth, and migration [168]. An in vivo study compared VIP924 to PV and LT in a humanized mouse model of MCL. Results demonstrated the superiority of VIP924 in suppressing tumor growth, improved survival, and tolerability. Notably, unlike LT, the animals in the VIP924 arm did not experience cytopenias [169]. These promising results support the need for further investigation in human clinical trials.

## 8. Future Directions of ADCs

Recent advances have been made in designing site-specific ADCs, utilizing biparatropic antibodies, and modifying cleavable linkers in efforts to make ADCs more efficient and effective, while minimizing toxicity.

A novel approach to conjugation chemistry involves site-specific attachment, which in some technologies utilizes the natural interchain disulfide bonds in antibodies, while other technologies require bioengineering to incorporate enzymatic conjugation tags into the antibody sequence [170]. Although it is yet to be explored in lymphoma, site-specific conjugation has been evaluated in solid malignancies such as breast, ovarian, and pancreatic cancer. One such application is the ThioMab technology, which involves site-specific conjugation using engineered cysteine. An ADC generated using this technology includes the anti-MUC16 ADC, which consists of inserting cysteine residues in specific positions on the light and heavy chain of trastuzumab, which are then coupled to a sulfhydryl group on cysteine with MMAE [171]. Additionally, the pClick technology provides site-specific conjugation; however, it utilizes native antibodies and a proximity-activated crosslinker without involving any bioengineering [172]. The ThioBridge technology consists of a cleavable linker that also utilizes native antibodies and consists of a three-carbon bridge attachment for enhanced stability [170,173]. Moreover, a recently innovated technology with the AJICAP second generation enables the production of site-specific ADCs without the aggregation challenges via utilization of selective cleavage reactions with thioester chemistry, and holds great promise for site-specific ADC without antibody engineering [174]. Lastly, tag-free enzymatic modification of native antibodies through different approaches such as glycan remodeling, transglutaminase, or lipoic-acid ligase approach holds promise as potential tools to advance the development of ADCs [175].

A promising class of ADCs are those that consist of a bivalent biparatopic antibody, as demonstrated in the HER-2 targeting ADC. The biparatopic antibody targets two non-overlapping epitopes, subsequently inducing receptor clustering and internalization. Such ADCs have demonstrated efficacy in breast cancer and preclinical studies examining MET-overexpressing tumors, offering potential for efficient cytotoxicity [176].

As for recent advances in linkers, efforts are focused on manufacturing linkers that are stable to avoid off-target payload release but also easily cleavable to deliver at the target site effectively. This leads to manufacturing linkers with pH and microenvironment-dependent cleavage, allowing ADCs to release the payload inside the cell [177].

Such advances are in development or preclinical phase studies, with a promise for improved therapeutic efficacy in the next generation of ADCs in various cancers including lymphomas.

## 9. Conclusions

ADCs are novel approaches that have revolutionized the treatment of Hodgkin and non-Hodgkin lymphomas. Advances of antibodies, linkers, and the payload will lead to further development of ADCs with higher specificity and cytotoxicity against lymphomas. While acquisition of resistance will be a limiting factor, combinational therapies with other novel agents, such us monoclonal antibodies, molecular targeted therapies, and bispecific antibodies will pave the way for new breakthrough treatments. There is an urgent need for innovative phase I studies to better understand the role of ADCs in different disease settings as we aim to increase the cure rates of lymphoid malignancies.

## Figures and Tables

**Table 1:** FDA-approved ADCs for lymphoma treatment.

ADC	Trade Name	Company	FDA Approval Lymphomas	Target Antigen	Year of Initial FDA Approval
Brentuximab Vedotin	ADCETRIS^®^	Seagen Genetics, Millennium/Takeda	cHL, CD30+T cell lymphomas	CD30	2011
Polatuzumab Vedotin	POLIVY^®^	Genentech, Roche	DLBCL	CD79B	2019
Loncastuximab Tesirine	ZYNLONTA^®^	ADC Therapeutics	DLBCL	CD19	2021

Abbreviations: PTCL: Peripheral T-Cell Lymphoma, cHL: Classic Hodgkin Lymphoma.

## Data Availability

Data sharing is not applicable to this article as no new data were created or analyzed in this study.
